# Physiotherapy for the Management of Polymyalgia Rheumatica: Results From a UK Cross‐Sectional Survey

**DOI:** 10.1002/msc.70155

**Published:** 2025-07-06

**Authors:** Anne V. O'Brien, Sara Muller, Jennifer Liddle, Martin J. Thomas, Christian D. Mallen

**Affiliations:** ^1^ School of Allied Health Professions and Pharmacy Keele University Staffordshire UK; ^2^ Primary Care Centre Versus Arthritis School of Medicine Keele University Staffordshire UK; ^3^ Population Health Sciences Institute Newcastle University Newcastle upon Tyne UK; ^4^ Haywood Academic Rheumatology Centre Midlands Partnership University NHS Foundation Trust Haywood Hospital Staffordshire UK; ^5^ Faculty of Medicine and Health Sciences Keele University Staffordshire UK

**Keywords:** exercise, patient education, physiotherapy, PMR, polymyalgia rheumatica, survey questionnaire

## Abstract

**Introduction:**

Polymyalgia rheumatica (PMR) international management guidelines advocate patient education and individualised exercises but lack evidence and physiotherapy practice for PMR is unknown. PMR is typically treated with glucocorticoids, but side effects are frequent and concerning to patients. This study investigated UK physiotherapy practice in PMR.

**Method:**

Physiotherapists recruited from UK rheumatology and physiotherapy professional networks and university alumni were invited to complete a postal or online questionnaire. Topics included experiences of managing PMR, perceived role and value of physiotherapy in PMR, assessment and management priorities and physiotherapists' education about PMR. Results were summarised using descriptive statistics.

**Results:**

4288 invitations to participate were sent, and 1072 (25%) responses were received. Physiotherapy referrals for PMR were infrequent; 5.8% of respondents had treated ≥ 10 patients in the previous year. 80% of respondents advocated a physiotherapy role for PMR. 38% reported receiving some pre‐registration education about PMR within their qualifying physiotherapy programme. Establishing patients' knowledge and understanding of PMR, pain levels, and ability to undertake activities of daily living were physiotherapists' assessment priorities. 90% of respondents promoted self‐management approaches, including pacing and activity modification. Prioritising upper limbs, 89% prescribed individualised graded exercises to improve movement, muscle strength and activities of daily living function.

**Conclusion:**

A positive role for physiotherapy was reported for some people with PMR. Exercise, education and advice to improve daily functioning may be useful adjuncts to glucocorticoids. The limited PMR education for UK physiotherapists warrants attention. Further research is needed to evaluate the effectiveness of physiotherapy approaches for PMR.

## 1 Introduction

Polymyalgia rheumatica (PMR) is one of the most common inflammatory rheumatic disorders affecting older people. With increased life expectancy and later retirement age (Office for National Statistics 2022; Chatzigeorgiou and Mackie 2018), the impact of PMR is likely to increase, including in working populations. The UK incidence of PMR is 95.9 per 100,000 person years, with an estimated lifetime prevalence of 2.4% for females and 1.7% for males (Partington et al. 2018; Crowson and Matteson 2017; Crowson et al. 2011).

Typically, people present with a rapid onset of bilateral shoulder and hip girdle pain (Mackie et al. 2014) and complain of stiffness, often enduring throughout the day (Muller et al. 2016; Mackie et al. 2015). Symptoms may significantly impact daily functioning and quality of life (Harris et al. 2023; Cawley et al. 2018; Dejaco et al. 2015; Twohig et al. 2015). Restricted overhead activities such as dressing or combing hair are common everyday difficulties reported by people with PMR (Mackie et al. 2015), whilst hip girdle pain can impact walking, getting on and off a toilet, turning over in bed and sexual function (Muller et al. 2022; Twohig et al. 2015).

Most people with PMR respond rapidly to initial glucocorticoid treatment (Dejaco et al. 2015; Mackie and Pease 2013; Matteson et al. 2012), but tapering to stop treatment is not always successful and can be protracted (Partington et al. 2018; Shbeeb et al. 2018; Dejaco et al. 2015; Matteson et al. 2012; Hutchings et al. 2007). For some people, symptoms persist and there is an associated treatment burden with prolonged prescriptions; ≥ 50% report significant side effects with glucocorticoids (Dejaco et al. 2015; Mazzantini et al. 2012), negatively impacting their quality of life (Harris et al. 2023; Hoon et al. 2019). Potential side effects include bone density loss leading to fragility fractures and osteoporosis, hypertension, diabetes, myopathy, mood disturbance, fatigue and weight gain (British National Formulary 2025; Mazzantini et al. 2012). As well as potentially profound consequences on morbidity (National Institute for Health and Care Excellence 2021; Hoes et al. 2009), patients with PMR report these side effects to be concerning (Harris et al. 2023; Hoon et al. 2019; Black et al. 2017; Muller et al. 2018). Alternative treatments in PMR have been identified as a research priority by guideline authors (Dejaco et al. 2015) and also by patients (Harris et al. 2023). Non‐pharmacological therapies, including exercise, have been reported to be commonly used by people with PMR in the UK (Weddell et al. 2022; Muller et al. 2018) but have not been evaluated.

International PMR management guidelines (Dejaco et al. 2015) recommend the consideration of individualised exercises to maintain muscle mass and function, especially for older people living with frailty. More specifically, balance and strengthening exercises are suggested prophylactically to reduce the risk of falls as well as the subsequent associated adverse events (including fractures) related to glucocorticoid use.

Although both patient priorities and international guidelines suggest consideration of physiotherapy interventions, there is an acknowledged potential for such interventions to also improve independence and mental wellbeing for older people (National Institute for Health and Care Excellence 2016). However, there is no established evidence base for the use of physiotherapy in the care of patients with PMR.

The objective of this study was to describe UK physiotherapists' experiences and opinions on the role of physiotherapy for PMR to support the further development of future interventions for rigorous evaluation.

## 2 Method

### 2.1 Study Design

A cross‐sectional survey was designed with both postal and online response options. The questionnaire content was informed by expert opinions from clinical and academic physiotherapy networks and published PMR research priorities (Dejaco et al. 2015). Three people with lived experience of PMR contributed to the survey design, reviewed initial questions and verified that the content was relevant to their experiences. The questionnaire was piloted with four physiotherapists specialising in musculoskeletal/rheumatology conditions. Minor rewording was recommended before the survey was finalised.

### 2.2 Participants

UK Health and Care Professions Council (HCPC) registered physiotherapists with self‐declared musculoskeletal experience were contacted and invited to respond to the questionnaire. Participant recruitment was informed by previous UK physiotherapy practice survey questionnaires (Grieve and Palmer 2017; Bishop et al. 2016). Potential participants were selected using the UK national rheumatology professional network; the British Society for Rheumatology (BSR), the UK National Rheumatoid Arthritis Society (NRAS) and National Axial Spondyloarthritis Society (NASS) (both of which are leading UK charities supporting people living with inflammatory musculoskeletal (MSK) conditions) both open to physiotherapy members, the Acupuncture Association of Chartered Physiotherapists, a special interest group for UK physiotherapists, National Institute for Health and Care Research (NIHR) physiotherapy clinical research networks, and physiotherapy alumni from Keele University (Table 1).

**TABLE 1:** Response by recruiting physiotherapy network.

Recruitment network and response method	Invited (*n*)	Responded *n* (%)
Postal response	(3727)	(1059)
Professional networks^a^	286	84 (29.4)
Acupuncture association of chartered physiotherapists	2979	804 (27.0)
Physiotherapy clinical research networks	462	171 (37.0)
Online response		
Online SurveyMonkey	561	32 (5.7)
Total respondents (duplicates removed, n19)	4288	1072 (25%)

^a^ British Society of Rheumatology/National Rheumatoid Arthritis Society/National Axial Spondyloarthritis Society/School of Allied Health Professions, Keele University physiotherapy alumni.

### 2.3 Procedures

Where email addresses were known (*n* = 561), potential respondents were emailed a participant information leaflet, consent form and an online link to the survey questionnaire designed using the SurveyMonkey (https://www.surveymonkey.com) platform. Paper‐based versions with pre‐paid reply envelopes were sent to physiotherapists whose postal addresses were known by professional networks. To protect the personal information of individual members, UK professional organisations (BSR, NRAS, NASS) sent invitation packs (participant information leaflet, consent form, postal survey questionnaires) to members directly in the summer of 2015.

Reminder postcards and a second copy of the study pack were sent to all postal non‐responders after two and 4 weeks, respectively. Automated alert reminders were similarly sent by email to online non‐responders. Anticipating that some physiotherapists could be members of multiple professional organisations, respondents' date of birth, sex and year of initial qualification were used to identify and remove any potential duplicate responses. Only the first responses were used in the analysis (*n* = 1072).

### 2.4 Data Analysis

Descriptive statistics (proportions of responses in percentages; means (SD), median (IQR)) were used to report survey findings. SPSS 22 [IBM Corp. Released in 2013. IBM Statistics for Windows, Version 22.0. Armonk, NY: IBM Corp.] was used for all analyses.

**Ethical approval** was obtained from Keele University Research Ethics Committee Ref: ERP2239 (January 2015), West Midlands Research Delivery Network (formerly Clinical Research Network) National Health Service (NHS) Assurance Review IRAS Ref: 169062 (February 2015) and NHS Research and Development Ethics for England and devolved nations (MREC Ref: NRS15/PY02).

## 3 Results

**FIGURE 1:**
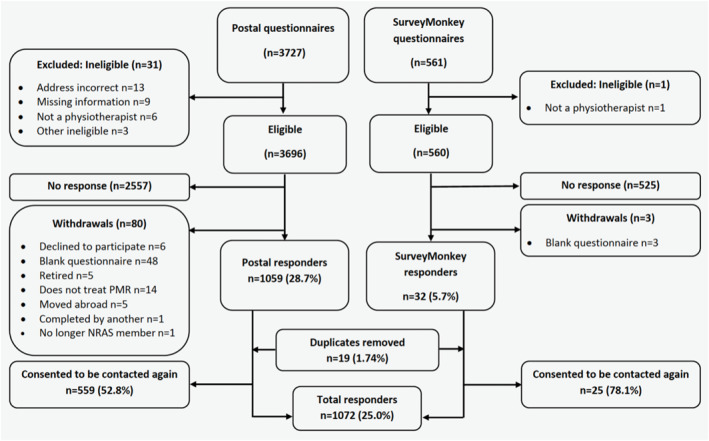
Flow diagram of respondents with reasons for exclusion of data. NRAS, National Rheumatoid Arthritis Society; PMR, polymyalgia rheumatica.

Details of those who responded from different professional networks are presented in Table 1. 4288 invitations were sent (3727 by post, 561 online) and once identified duplicates (*n* = 19) were removed. 1072 (25%) responses were included in the final analysis. Reasons for exclusion are reported in Figure 1. Respondents were 81.8% female; their median age was 44.0 years (36‐52 IQR) and they were experienced physiotherapists with a median of 18.0 (4‐29 IQR) years since qualifying (Table 2). Physiotherapists working across multiple settings were common; 445 (42.5%) respondents reported their employment to be partially or fully within private practice, whereas 388 (37.1%) reported working in secondary care and 329 (31.5%) in primary care.

**TABLE 2:** Survey questionnaire respondent demographics.

Demographic category	All respondents (*n* = 1072)
Age median (IQR)	44.0 (36–52)
Years qualified median (IQR)	18.0 (4–29)
Male (%)	195 (18.2)
Declared interest in inflammatory arthritis (%)	187 (17.7)

NHS pay banding/Grade (*n* = 1046)	(*n*, %)
Band 5 (newly qualified)	28 (2.7)
Band 6	276 (26.4)
Band 7 (senior specialist staff)	310 (29.6)
Band 8a or 8b (Consultant physiotherapists)	120 (11.5)
Unstated/not applicable^a^	312 (29.8)

Original physiotherapy qualification (*n* = 1034)	(*n*, %)
BSc (Hons) physiotherapy	662 (64.0)
Graduate diploma physiotherapy	339 (32.8)
Pre‐registration MSc physiotherapy	27 (2.6)
Remedial gymnast/qualified overseas	6 (0.6)

Postgraduate studies	(*n*, %)
Undertaken one or more masters' modules	275 (33.9)
Completed an MSc degree	182 (22.4)
Completed an MPhil or doctorate	25 (3.1)

Physiotherapists' experience of PMR in year prior to survey completion	(*n*, %)
Not seen any patients	297 (27.8)
1–5 patients	623 (58.2)
6–10 patients	88 (8.2)
> 10 patients	62 (5.8)

Source of PMR knowledge	(*n*, %)
Pre‐registration physiotherapy programme	387 (38.3%)
Post‐registration PMR‐related education	256 (24.8%)

Confidence progressing people with PMR	(*n*, %)
Always confident	419 (41.5%)
Sometimes confident	503 (50.0%)
Rarely confident	71 (7.1%)
Never confident	14 (1.4%)

Abbreviation: IQR, interquartile range.

^a^ Not working in NHS.

### 3.1 Physiotherapists' Experience and Belief in Role Working With People With PMR

Physiotherapists' experience of working with people with PMR was limited. 62 (5.8%) respondents had treated more than 10 patients with PMR in the previous year (Table 2), with physiotherapists working in secondary care (hospital) settings reporting the most experience. Despite acknowledged limited exposure to PMR, 846 (80%) respondents categorically advocated for a role for physiotherapy to support people with PMR and 198 (18.7%) reported that there “may” be a role.

### 3.2 Physiotherapy Assessments and Priorities

**FIGURE 2:**
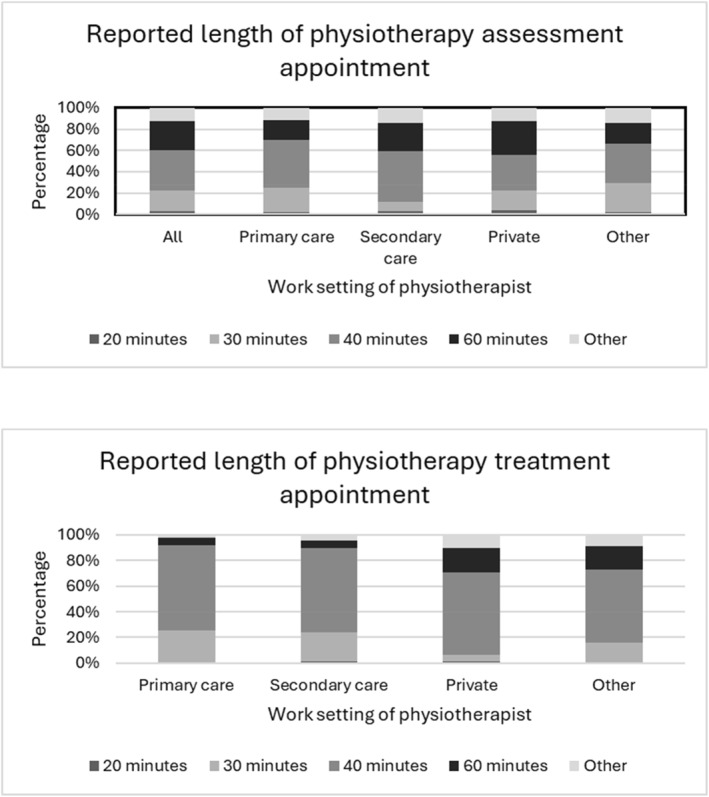
Lenth of appointments for physiotherapy assessment and treatment by work setting.

The duration of initial physiotherapy assessment appointments varied across work settings but was typically 40 min (Figure 2). Physiotherapy assessment priorities were broadly the same regardless of work setting (Table 3). However, assessing pain was considered more important by respondents working in private practice settings (284, 61.9%) than those in primary (210, 55.5%) and secondary care (164, 50.2%). However, assessing the patient's knowledge and understanding of PMR was similar, although considered slightly more of a priority by physiotherapists working in primary (242, 63.7%) and secondary (206, 63.8%) care NHS settings than private practice (273, 59.0%).

**TABLE 3:** Physiotherapy assessment priorities for PMR by work setting.^a^

Assessment priorities	All work settings^b^ *n* (%)	Primary care^b^ *n* (%)	Secondary care^b^ *n* (%)	Private practice^b^ *n* (%)	Other setting^c^ ^,^ ^b^ *n* (%)
PMR knowledge	633 (60.0)	242 (63.7)	206 (63.8)	273 (59.0)	273 (57.0)
Pain	614 (58.4)	210 (55.4)	164 (50.2)	284 (61.9)	284 (60.3)
Activities of daily living	556 (52.5)	191 (50.1)	170 (52.3)	249 (53.2)	249 (51.2)
Pacing	400 (37.9)	149 (39.2)	120 (37.2)	183 (39.4)	183 (30.2)
General mobility	279 (26.3)	101 (26.4)	84 (25.7)	115 (24.7)	115 (26.9)
Fatigue	127 (22.0)	51 (13.4)	36 (11.0)	63 (13.5)	63 (13.0)
Joint range of movement	182 (17.2)	67 (17.6)	67 (20.4)	70 (15.0)	70 (19.4)
Early morning stiffness	101 (9.5)	35 (9.2)	32 (9.8)	50 (10.7)	50 (7.6)
Muscle strength	76 (7.2)	32 (8.4)	30 (9.2)	33 (7.1)	33 (5.5)
Functional tests	35 (3.3)	13 (3.4)	13 (4.0)	20 (4.3)	20 (5.0)

^a^ Ranked 1–3 or checked as an important priority *n* (%).

^b^ Multiple answers for work settings were permitted.

^c^ Other work setting: University, Industry, Intermediate Care, Community/Domiciliary, Accident & Emergency, Chronic pain services, Oncology, Neurological rehabilitation, Paediatrics, Obstetrics and Gynaecology/Women's Health, or Sports Medicine work settings.

UK physiotherapists ranked assessment priorities as follows: establishing patients' knowledge and understanding of PMR (633, 60.0%), evaluating pain (614, 58.4%) and determining a patient's ability to undertake activities of daily living (ADLs) (522, 52.5%) (Table 3). These priorities were similarly identified, regardless of work setting, experience of caring for patients with PMR, or reported belief in a physiotherapy role in PMR management.

### 3.3 Influence of Glucocorticoid Dosage on Physiotherapy Management

Respondents also reported that physiotherapy assessments typically included a full social and medication history. Specifically, 981 respondents (95.5%) always established if patients with PMR were taking glucocorticoid medication, 860 (83.8%) ascertained how long the patient had been taking these medications and 621 (60.8%) established the precise dose. 409 responders (40.0%) were able to access laboratory results. Of these, 119 (27.5%) respondents stated these results “always” or 245 (56.6%) “sometimes” impacted their physiotherapy management of the patient.

### 3.4 Physiotherapy Management

Managing pain via varied strategies was a priority for physiotherapists (Table 4). Local heat treatments using hot packs, warm showers or hydrotherapy were preferred over cold therapy. Acupuncture was more likely to be endorsed by those working in private practice and was reported to be used more frequently for pain relief (341, 33.3%) than transcutaneous electrical nerve stimulation (235, 23.1%).

**TABLE 4:** Specific pain management therapeutic modalities used by physiotherapists.

Pain modality	*n* (%) (total respondents 1072)			
	Yes	Maybe	No	Not answered
Hydrotherapy	396 (36.9)	472 (44.0)	140 (13.1)	64 (6.0)
Heat	363 (33.9)	533 (49.7)	115 (10.7)	61 (5.7)
Acupuncture	341 (31.8)	500 (46.6)	183 (17.1)	48 (4.5)
TENS	235 (21.9)	591 (55.2)	192 (17.9)	54 (5.0)
Cold therapy	123 (11.5)	533 (49.7)	355 (33.1)	61 (5.7)

Abbreviation: TENS, transcutaneous electrical nerve stimulation.

Promoting self‐management via patient education was specifically identified as a key element of physiotherapy treatment by 90.1% of respondents. Pacing advice and exercise therapy were the most frequently reported self‐management strategies (Table 5). 58% of respondents reported supplementing educational advice given during consultations with written information.

**TABLE 5:** Pain‐related self‐management strategies^a^ used by UK physiotherapists for PMR.

Pain self‐management strategy promoted by physiotherapists	*n* (%) (respondents 1072)
Pacing	976 (91)
Exercise	954 (89)
Analgesia	761 (71)
Work advice	708 (66)
Joint protection advice	590 (55)
Energy conservation	450 (42)

^a^ Multiple responses were permitted.

### 3.5 Exercise Therapy in PMR

Physiotherapists have reported prescribing exercises for multiple reasons. When asked about the purpose of prescribing exercise in PMR, 781 (76.6%) respondents reported improving ADL function was their intention; 760 (75.0%) aimed to improve joint range and 659 (67.7%) aimed to improve muscle strength. Limb girdles were emphasised more in exercise prescription than the spine; 730 (68.1%) respondents included exercises targeted for the shoulder girdle, whilst 575 (53.6%) of physiotherapists included the pelvic girdle. 702 respondents (65%) reported they might prescribe proprioception and balance exercises, while 652 (61%) reported they might prescribe weight bearing exercises. The least likely exercise strategy to be prescribed was for cardiovascular conditioning, reported by 49.1% (526) of respondents.

### 3.6 Treatment Duration and Follow‐Up

Treatment appointments were typically shorter than initial assessments for people with PMR; 638 respondents (62.9%) reported taking a 30‐min appointment for each PMR physiotherapy treatment session. Private practitioners, however, allocated more time per treatment than physiotherapists working in other settings. Across all employment settings, around half of the patients were discharged within 3 months of their first appointment (Figure 2).

### 3.7 Physiotherapy Outcome Measures

Physiotherapists used outcome measures inconsistently. Less than half of respondents (*n* = 422; 43.6%) reported “always” using an outcome measure, 241 (24.9%) “sometimes” included one in their physiotherapy assessment. Various domains were evaluated, but assessment of pain, functional ability, and establishing quality of life living with PMR were the most frequently reported outcome measures used.

## 4 Discussion

This study suggests that UK physiotherapists infrequently treat people living with PMR but believe that there is a role for physiotherapy for people with PMR. Key assessment priorities were PMR knowledge, pain and ADL function. The reported promotion of self‐management options, typically via individualised and targeted exercise prescriptions, and patient education were focused to improve ADL function.

Survey respondents were a broad spectrum of UK MSK physiotherapists and broadly representative of the physiotherapy population at the time of survey completion in 2015 (Chartered Society of Physiotherapy 2016). Respondents represented a range of experience treating people with PMR, although overall levels of physiotherapists' self‐reported experience of PMR were limited, especially compared with other MSK conditions (Grieve and Palmer 2017; Bishop et al. 2016). Infrequent exposure to PMR has also been reported by UK doctors working in primary care; a fulltime General Practitioner (GP) may only see five patients with PMR in a typical year (Helliwell et al. 2013), which might go some way to explaining to lack of satisfaction reported by some patients (Muller et al. 2018).

Physiotherapists have reported using exercise to enhance patient independence when undertaking ADLs by regaining range of movement and increasing muscle strength. This is in keeping with the physiotherapy management of other MSK (some inflammatory) conditions (National Institute for Health and Care Excellence 2022; O'Shea et al. 2022; National Institute for Health and Care Excellence 2017, 2020). This study has shown that balance and strengthening exercises are also prescribed by some physiotherapists for people with PMR. Including such exercises is in keeping with others who also promote regular tailored weight‐bearing exercises for post‐menopausal women and people at risk of fragility fracture (The National Osteoporosis Guideline Group 2021; Stanghelle et al. 2020), such as those receiving long‐term glucocorticoid treatment. As most people being treated for PMR take over 5 mg of prednisolone for more than 3 months (Paskins et al. 2018), this approach is logical although its effectiveness is not known in this population. However, Barker et al. (2020) suggest that exercises to reduce fracture risk and other fall‐related injuries may help to maintain people's independence and reduce related healthcare burdens on the individual, their families and society.

The infrequency of physiotherapy referral for PMR management is another new finding identified in this study. In the absence of national referral data, anecdotally, this is thought to be notably less frequent than in other inflammatory and MSK conditions. The relatively low prevalence of PMR compared with other MSK conditions affecting older people (e.g. osteoarthritis) is one obvious factor, but this low referral rate may simply reflect a lack of appreciation of the potential for physiotherapy in PMR from primary care practitioners. Regardless, the reasons are unknown but likely to be multi‐factorial. The perceived effectiveness of glucocorticoids in PMR may be another factor, although data show that the patient experience of symptom relief and treatment tapering is variable (Partington et al. 2018; Shbeeb et al. 2018; Hutchings et al. 2007). Peoples' experience living with PMR also varies significantly (Muller et al. 2020). Cawley et al. (2018) reported that some people with PMR experience minimal functional loss, while others describe significant difficulties impacting daily life, even “a life‐changing reduction in their ability to carry out many activities of daily living” (Twohig et al. 2018). The fact that PMR is largely managed in primary care (Yates et al. 2016; Dasgupta et al. 2012) with only a minority of patients referred into rheumatology specialist services may also be relevant, as might the fact that in other MSK conditions, for example osteoarthritis and rheumatoid arthritis, clinical guidelines explicitly promote a role for physiotherapy (National Institute for Health and Care Excellence 2020, 2022) and have an established evidence base, whereas this is new research territory in PMR.

This study identified that UK physiotherapists believe that physical functioning and self‐efficacy can be improved by physiotherapy for people living with PMR. Multiple combinations of patient education and advice were reported. But this is not surprising in the absence of any empirical evidence proposing optimal content of patient education or therapeutic exercise programmes, or guidance indicating who might benefit the most from such intervention. It is also currently unclear when any physiotherapy intervention may be more effective, for example possibly following a PMR diagnosis or flare. The optimal duration for physiotherapy treatment, the length of any positive impact and the cost‐effectiveness of physiotherapy interventions all potentially warrant further investigation. Given the life‐changing impact of PMR in some people “a life‐changing reduction in their ability to carry out many activities of daily living” and “no‐one gets back to feeling as well as before” (Twohig et al. 2018), coupled with patients wanting prioritisation of research into alternatives to glucocorticoid treatment, suggests it may now be timely to investigate the potential benefits of physiotherapy for PMR. However, the relatively low numbers of patients in each primary care centre, small proportion referred to rheumatology and infrequent referrals to physiotherapy might make the design and conduct of future studies, particularly trials of complex interventions, more challenging.

There are several limitations to this study. First, data were collected before the coronavirus pandemic and the mode of delivery for some physiotherapy services has changed since then. In particular, some MSK physiotherapy consultations have been adapted to enable digital health appointments (NHS England 2023; Greenhalgh et al. 2020). However, exercise therapy, patient education and self‐management strategies remain core to contemporary MSK physiotherapy, so the essence of physiotherapy for PMR is unlikely to have substantially changed. Second, the 25% survey response rate was relatively low. Earlier UK MSK physiotherapy surveys have reported higher response rates (Holden et al. 2019; Bishop et al. 2016), but were related to more prevalent conditions which are more frequently treated by physiotherapists, which might therefore elicit a higher response rate. Although over 1000 responses were received, a large proportion (43%) of these physiotherapists worked, even partially, in private practice (Moffatt et al. 2024; Grieve and Palmer 2017; Bishop et al. 2016). This might simply reflect the sampling frame for the study, which included members of the UK Acupuncture Association of Chartered Physiotherapists. This study only reflects UK physiotherapy practice; therefore, it is not known if PMR physical therapy approaches in other countries would be comparable. Third, it is unclear to what extent self‐declared MSK physiotherapists might deem there to be a role for their own profession in the management of any condition (such as PMR) that is inherently musculoskeletal, even though they receive little education about PMR. Therefore, it will be important in future to consider the views of other interested parties and particularly patients. Whilst desiring alternatives to glucocorticoid treatment and better information about PMR, patients would have to actively engage and partake in physiotherapy exercises and self‐management approaches. Further work is therefore required to understand whether this would be acceptable to patients or whether patients would prefer more passive treatment options.

## 5 Conclusion

This study provides the first description of reported physiotherapy practice for people with PMR. It has highlighted that UK physiotherapists believe they can fulfil a valuable role supporting people with PMR to improve functioning and to self‐manage their condition, despite receiving few referrals to assess people with PMR and having little pre‐ and post‐registration PMR education. Demographic shifts mean that the number of people living with PMR and working into older age is likely to increase. As such, it is timely to evaluate the potential role of physiotherapy interventions in PMR. As an adjunct to glucocorticoid treatment, physiotherapy could help improve patients' quality of life and potentially decrease the medication burden.

## Author Contributions

Study concept and design: Anne V. O'Brien and Christian D. Mallen. Acquisition of data: Anne V. O'Brien. Analysis and interpretation of data: Anne V. O'Brien, Sara Muller and Jennifer Liddle. Drafting of the manuscript: Anne V. O'Brien. Critical revision of the manuscript: Sara Muller, Christian D. Mallen, Martin J. Thomas and Jennifer Liddle. Study supervision: Sara Muller, Christian D. Mallen, Jennifer Liddle and Martin J. Thomas.

## Ethics Statement

Ethical approval was obtained from Keele University Research Ethics Committee Ref: ERP2239 (January 2015), West Midlands Research Delivery Network (formerly Clinical Research Network) National Health Service (NHS) Assurance Review IRAS Ref: 169062 (February 2015) and NHS research and development ethics for England and devolved nations (MREC Ref: NRS15/PY02).

## Conflicts of Interest

The authors declare no conflicts of interest.

## Supporting information

Supporting Information S1

## Data Availability

The data that support the findings of this study are available from the corresponding author upon reasonable request.

## References

[msc70155-bib-0001] Barker, K. L. , M. Newman , N. Stallard , et al. 2020. “Physiotherapy Rehabilitation for Osteoporotic Vertebral Fracture—A Randomised Controlled Trial and Economic Evaluation (PROVE Trial).” Osteoporosis International 31, no. 2: 277–289. 10.1007/s00198-019-05133-0. 31720722

[msc70155-bib-0002] Bishop, A. , M. A. Holden , R. O. Ogollah , and N. E. Foster . 2016. “Current Management of Pregnancy‐Related Low Back Pain: A National Cross‐Sectional Survey of UK Physiotherapists.” Physiotherapy 102, no. 1: 78–85. 10.1016/j.physio.2015.02.003. 26050136

[msc70155-bib-0003] Black, R. J. , S. M. Goodman , C. Ruediger , S. Lester , S. L. Mackie , and C. L. Hill . 2017. “A Survey of Glucocorticoid Adverse Effects and Benefits in Rheumatic Diseases: The Patient Perspective.” Journal of Clinical Rheumatology 23, no. 8: 416–420. 10.1097/rhu.0000000000000585. 28926469

[msc70155-bib-0004] British National Formulary 2025. National Institute for Clinical Excellence, Glucocorticoids: General Use. Corticosteroids, general use | Treatment summaries | BNFC | NICE. Accessed 3.6.25.

[msc70155-bib-0005] Cawley, A. , J. A. Prior , S. Muller , et al. 2018. “Association Between Characteristics of Pain and Stiffness and the Functional Status of Patients With Incident Polymyalgia Rheumatica From Primary Care.” Clinical Rheumatology 37, no. 6: 1639–1644. 10.1007/s10067-017-3730-6. 28634698 PMC5948286

[msc70155-bib-0006] Chartered Society of Physiotherapy . 2016. Membership Data. http://www.csp.org.uk/documents/crm‐report‐%E2%80%93‐membership‐data‐13.

[msc70155-bib-0007] Chatzigeorgiou, C. , and S. L. Mackie . 2018. “Comorbidity in Polymyalgia Rheumatica.” Reumatismo 70, no. 1: 35–43. 10.4081/reumatismo.2018.1039. 29589401

[msc70155-bib-0008] Crowson, C. S. , and E. L. Matteson . 2017. “'Contemporary Prevalence Estimates for Giant Cell Arteritis and Polymyalgia Rheumatica, 2015.” Seminars in Arthritis and Rheumatism 42, no. 2: 253–256. 10.1016/j.semarthrit.2017.04.001. PMC5623160 28551169

[msc70155-bib-0009] Crowson, C. S. , E. L. Matteson , E. Myasoedova , et al. 2011. “The Lifetime Risk of Adult‐Onset Rheumatoid Arthritis and Other Inflammatory Autoimmune Rheumatic Diseases.” Arthritis & Rheumatism 63, no. 3: 633–639. 10.1002/art.30155. 21360492 PMC3078757

[msc70155-bib-0010] Dasgupta, B. , M. A. Cimmino , H. M. Kremers , et al. 2012. “2012 Provisional Classification Criteria for Polymyalgia Rheumatica: A European League Against Rheumatism/American College of Rheumatology Collaborative Initiative.” Arthritis & Rheumatism 64, no. 4: 943–954. 10.1136/annrheumdis-2011-200329. 22389040

[msc70155-bib-0011] Dejaco, C. , Y. P. Singh , P. Perel , et al. 2015. “2015 Recommendations for the Management of Polymyalgia Rheumatica: A European League Against Rheumatism/American College of Rheumatology Collaborative Initiative.” Annals of the Rheumatic Diseases 74, no. 10: 1799–1807. 10.1136/annrheumdis-2015-207492. 26359488

[msc70155-bib-0012] Greenhalgh, S. , L. Finucane , C. Mercer , and J. Selfe . 2020. “Safety Netting; Best Practice in the Face of Uncertainty.” Musculoskeletal Science and Practice 48: 1–3. 10.1016/j.msksp.2020.102179. PMC7214294 32560875

[msc70155-bib-0013] Grieve, R. , and S. Palmer . 2017. “'Physiotherapy for Plantar Fasciitis: A UK‐Wide Survey of Current Practice.” Physiotherapy 103, no. 2: 193–200. 10.1016/j.physio.2016.02.002. 27156704

[msc70155-bib-0014] Harris, G. K. , J. L. Leung , and R. R. Buchanan . 2023. “Exploring the Patient Experience in Polymyalgia Rheumatica.” Clinical Rheumatology 42, no. 12: 3421–3422. 10.1007/s10067-023-06794-3. 37861872

[msc70155-bib-0015] Helliwell, T. , S. L. Hider , and C. D. Mallen . 2013. “Polymyalgia Rheumatica: Diagnosis, Prescribing, and Monitoring in General Practice.” British Journal of General Practice: Journal of the Royal College of General Practitioners 63, no. 610: e361–e366. 10.3399/bjgp13X667231. PMC3635583 23643235

[msc70155-bib-0016] Hoes, J. N. , J. W. Jacobs , S. M. Verstappen , J. W. Bijlsma , and G. J. Van der Heijden . 2009. “'Adverse Events of Low‐ to Medium‐Dose Oral Glucocorticoids in Inflammatory Diseases: A Meta‐Analysis.” Annals of the Rheumatic Diseases 68, no. 12: 1833–1838. 10.1136/ard.2008.100008. 19066177

[msc70155-bib-0017] Holden, M. A. , R. Whittle , J. Waterfield , et al. 2019. “A Mixed Methods Exploration of Physiotherapist's Approaches to Analgesic Use Among Patients With Hip Osteoarthritis.” Physiotherapy 105, no. 3: 328–337. 10.1016/j.physio.2018.08.003. 30318127

[msc70155-bib-0018] Hoon, E. , C. Ruediger , T. K. Gill , R. J. Black , and C. L. Hill . 2019. “A Qualitative Study of Patient Perspectives Related to Glucocorticoid Therapy in Polymyalgia Rheumatica and Giant Cell Arteritis.” Open Access Rheumatology Research and Reviews 11: 189–198. 10.2147/OARRR.S213964. 31695526 PMC6718238

[msc70155-bib-0019] Hutchings, A. , J. Hollywood , D. L. Lamping , et al. 2007. “Clinical Outcomes, Quality of Life, and Diagnostic Uncertainty in the First Year of Polymyalgia Rheumatica.” Arthritis & Rheumatism 57, no. 5: 803–809. 10.1002/art.22777. 17530680

[msc70155-bib-0020] Mackie, S. L. , S. Arat , J. da Silva , et al. 2014. “'Polymyalgia Rheumatica (PMR) Special Interest Group at OMERACT 11: Outcomes of Importance for Patients With PMR.” Journal of Rheumatology 41, no. 4: 819–823. 10.3899/jrheum.131254. 24488422

[msc70155-bib-0021] Mackie, S. L. , R. Hughes , M. Walsh , et al. 2015. “‘An Impediment to Living Life’: Why and How Should We Measure Stiffness in Polymyalgia Rheumatica?” PLoS One 10, no. 5: 1–13. 10.1371/journal.pone.0126758. PMC4425533 25955770

[msc70155-bib-0022] Mackie, S. L. , and C. T. Pease . 2013. “'Diagnosis and Management of Giant Cell Arteritis and Polymyalgia Rheumatica: Challenges, Controversies and Practical Tips.” Postgraduate Medical Journal 89, no. 1051: 284–292. 10.1136/postgradmedj-2012-131400. 23355687

[msc70155-bib-0023] Matteson, E. L. , H. Maradit‐Kremers , M. A. Cimmino , et al. 2012. “Patient‐Reported Outcomes in Polymyalgia Rheumatica.” Journal of Rheumatology 39, no. 4: 795–803. 10.3899/jrheum.110977. 22422492

[msc70155-bib-0024] Mazzantini, M. , C. Torre , M. Miccoli , et al. 2012. “Adverse Events During Longterm Low‐Dose Glucocorticoid Treatment of Polymyalgia Rheumatica: A Retrospective Study.” Journal of Rheumatology 39, no. 3: 522–527. 10.3899/jrheum.110851. 22247343

[msc70155-bib-0025] Moffatt, M. , S. Lalande , N. Maher , and C. Littlewood . 2024. “Rotator Cuff Disorders: An Updated Survey of Current (2023) UK Physiotherapy Practice.” Musculoskeletal Care 22, no. 1: 1–8. 10.1002/msc.1872. 38407393

[msc70155-bib-0026] Muller, S. , S. L. Hider , T. Helliwell , et al. 2016. “'Characterising Those With Incident Polymyalgia Rheumatica in Primary Care: Results From the PMR Cohort Study.” Arthritis Research and Therapy 18, no. 200: e1–e9. 10.1186/s13075-016-1097-8. PMC5015343 27605116

[msc70155-bib-0027] Muller, S. , S. L. Hider , P. Ranasinghe , et al. 2022. “The Impact of Polymyalgia Rheumatica on Intimate Sexual Relationships: Findings From the PMR Cohort Study.” Rheumatol Adv Pract 6, no. 3. 10.1093/rap/rkac070. PMC9479881 36133964

[msc70155-bib-0028] Muller, S. , A. V. O'Brien , T. Helliwell , et al. 2018. “Support Available for and Perceived Priorities of People With Polymyalgia Rheumatica and Giant Cell Arteritis: Results of the PMRGCAuk Members' Survey 2017.” Clinical Rheumatology 37, no. 12: 3411–3418. 10.1007/s10067-018-4220-1. 30066282

[msc70155-bib-0029] Muller, S. , R. Whittle , S. L. Hider , et al. 2020. “Longitudinal Clusters of Pain and Stiffness in Polymyalgia Rheumatica: 2‐Year Results From the PMR Cohort Study.” Rheumatology 59, no. 8: 1906–1915. 10.1093/rheumatology/kez533. 31742642 PMC7382596

[msc70155-bib-0030] National Institute for Health and Care Excellence . 2016. Mental Wellbeing and Independence for Older People. https://www.nice.org.uk/guidance/qs137/resources/mental‐wellbeing‐and‐independence‐for‐older‐people‐pdf‐75545424081349.

[msc70155-bib-0031] National Institute for Health and Care Excellence . 2017. Spondyloarthritis in over 16s: Diagnosis and Management. National Institute for Health and Care Excellence. https://www.nice.org.uk/guidance/ng65. 28350428

[msc70155-bib-0032] National Institute for Health and Care Excellence . 2020. Rheumatoid Arthritis in Adults: Management. National Institute for Health and Care Excellence. https://www.nice.org.uk/guidance/ng100.

[msc70155-bib-0033] National Institute for Health and Care Excellence . 2021. “Chronic Pain (Primary and Secondary) in over 16s: Assessment of All Chronic Pain and Management of Chronic Primary Pain.”. NICE Guideline NG193. https://www.nice.org.uk/guidance/ng193/chapter/Recommendations#managing‐chronic‐primary‐pain. 33939353

[msc70155-bib-0034] National Institute for Health and Care Excellence . 2022. Osteoarthritis in over 16s: Diagnosis and Management. https://www.nice.org.uk/guidance/ng226/chapter/recommendations#information‐and‐support. 36745715

[msc70155-bib-0035] NHS England . 2023. “Guide to Adopting Remote Consultations in Adult Musculoskeletal Physiotherapy Services.”. Available at: NHS England » Guide to Adopting Remote Consultations in Adult Musculoskeletal Physiotherapy Services.

[msc70155-bib-0036] Office for National Statistics 2022. National Population Projections: 2021‐based Interim. National population projections ‐ Office for National Statistics.

[msc70155-bib-0037] O'Shea, A. , J. Drennan , C. Littlewood , H. Slater , J. Sim , and J. G. McVeigh . 2022. “Barriers and Facilitators Related to Self‐Management of Shoulder Pain: A Systematic Review and Qualitative Synthesis.” Clinical Rehabilitation 36, no. 11: 1539–1562. 10.1177/02692155221108553. 35733369 PMC9515516

[msc70155-bib-0038] Partington, R. J. , S. Muller , T. Helliwell , C. D. Mallen , A. A. Sultan , and A. Abdul Sultan . 2018. “'Incidence, Prevalence and Treatment Burden of Polymyalgia Rheumatica in the UK over Two Decades: A Population‐Based Study.” Annals of the Rheumatic Diseases 77, no. 12: 1750–1756. 10.1136/annrheumdis-2018-213883. 30297332

[msc70155-bib-0039] Paskins, Z. , R. Whittle , A. A. Sultan , et al. 2018. “Risk of Fracture Among Patients With Polymyalgia Rheumatica and Giant Cell Arteritis: A Population‐Based Study.” BMC Medicine 16, no. 1: 1–9. 10.1186/s12916-017-0987-1. PMC5761155 29316928

[msc70155-bib-0040] Shbeeb, I. , D. Challah , S. Raheel , C. S. Crowson , and E. L. Matteson . 2018. “Comparable Rates of Glucocorticoid Associated Adverse Events in Patients With Polymyalgia Rheumatica and Comorbidities in the General Population.” Arthritis Care & Research 70, no. 4: 643–647. 10.1002/acr.23320. 28704600 PMC6475501

[msc70155-bib-0041] Stanghelle, B. , H. Bentzen , L. Giangregorio , A. H. Pripp , D. A. Skelton , and A. Bergland . 2020. “Effects of a Resistance and Balance Exercise Programme on Physical Fitness, Health‐Related Quality of Life and Fear of Falling in Older Women With Osteoporosis and Vertebral Fracture: A Randomized Controlled Trial.” Osteoporosis international 31, no. 6: 1069–1078. 10.1007/s00198-020-05398-w. 31925473

[msc70155-bib-0042] The National Osteoporosis Guideline Group (2021). Clinical Guideline for the Prevention and Treatment of Osteoporosis. Full Guideline | NOGG.

[msc70155-bib-0043] Twohig, H. , G. Jones , S. Mackie , C. D. Mallen , and C. Mitchell . 2018. “Assessment of the Face Validity, Feasibility and Utility of a Patient‐Completed Questionnaire for Polymyalgia Rheumatica: A Postal Survey Using the QQ‐10 Questionnaire.” Pilot and Feasibility Studies 4, no. 7: 7. 10.1186/s40814-017-0150-y. 28694986 PMC5501557

[msc70155-bib-0044] Twohig, H. , C. Mitchell , C. Mallen , A. Adebajo , and N. Mathers . 2015. “‘I Suddenly Felt I’d Aged’: A Qualitative Study of Patient Experiences of Polymyalgia Rheumatica (PMR).” Patient Education and Counselling 98, no. 5: 645–650. 10.1016/j.pec.2014.12.013. 25638304

[msc70155-bib-0045] Weddell, J. , S. L. Hider , C. D. Mallen , and S. Muller . 2022. “What Non‐pharmacological Treatments Do People With Polymyalgia Rheumatica Try: Results From the PMR Cohort Study.” Rheumatology International 42, no. 2: 285–290. 10.1007/s00296-021-05036-6. 34677651 PMC8800888

[msc70155-bib-0046] Yates, M. , K. Graham , R. A. Watts , and A. J. MacGregor . 2016. “The Prevalence of Giant Cell Arteritis and Polymyalgia Rheumatica in a UK Primary Care Population.” BMC Musculoskeletal Disorders 17, no. 1: 285. 10.1186/s12891-016-1127-3. 27421253 PMC4946178

