# Supplementary material for: Physiotherapy for the Management of Polymyalgia Rheumatica: Results From a UK Cross‐Sectional Survey
Source: Musculoskeletal Care. 2025 Jul 6;23(3):e70155. doi: 10.1002/msc.70155 (PMC12230277; doi:10.1002/msc.70155)
Supplement: Supplementary file 1 — Supporting Information S1 [file MSC-23-e70155-s001.docx]

Supplementary Table - Appendix 1 (if permitted)

***Duration of physiotherapy assessments, treatments by work setting*§ *and reported appointment times and duration of follow up***

| **Physiotherapy assessment sessions** | | | | | |
| --- | --- | --- | --- | --- | --- |
| **Respondent work settings** | **20 minutes n (%)** | **30 minutes**  **n (%)** | **40 minutes**  **n (%)** | **60 minutes**  **n (%)** | **Other ‡**  **n (%)** |
| **All work settings †**  (n=1033) | 29 (2.8) | 202 (19.6) | 391 (37.9) | 280 (27.1) | 131 (12.7) |
| **Primary Care** (n=375) | 6 (1.6) | 89 (23.7) | 168 (44.8) | 69 (18.4) | 43 (11.5) |
| **Secondary Care** (n=319) | 7 (2.2) | 59 (8.5) | 135 (42.3) | 78 (24.5) | 40 (12.5) |
| **Private practice** (n=454) | 17 (3.7) | 85 (18.7) | 153 (33.7) | 143 (31.5) | 56 (12.3) |
| **Other §**  (n=145) | 3 (2.1) | 39 (26.9) | 54 (37.2) | 29 (20%) | 20 (13.8) |
| **Physiotherapy treatment sessions** | | | | | |
| **Primary Care** (n=375) | 3 (0.8) | 92 (24.9) | 243 (65.9) | 22 (6.0) | 9 (2.4) |
| **Secondary Care** (n=319) | 3 (1.0) | 71 (22.8) | 204 (65.6) | 19 (6.1) | 14 (4.5) |
| **Private practice** (n=448) | 4 (0.9) | 25 (5.6) | 287 (64.1) | 84 (18.8) | 48 (10.7) |
| **Other §**  (n=145) | 1 (0.5 ) | 29 (15.5) | 107 (57.2) | 34 (18.2) | 16 (8.6) |
| **Time from 1^st^ appointment to physiotherapy discharge** | **Immediate discharge**  **n (%)** | **1-3 months**  **n (%)** | **3-6**  **months**  **n (%)** | **Never**  **discharged**  **n (%)** |  |
| **All work settings** | 78 (7.6) | 439 (43.7) | 169 (16.8) | 50 (5.0) |  |

**†** Work setting responses were not mutually exclusive

**‡** Other assessment times reported (e.g. varies, 45 minutes)

§ Other work settings included: Community, Intermediate Care, University and Industry
